# Spontaneous and enormous, chronic expanding hematoma of the lumbar region: a case report

**DOI:** 10.1186/1757-1626-2-9400

**Published:** 2009-12-24

**Authors:** Dritan Pasku, Artan Bano, Eleni Lagoudaki, Kalliopi Alpantaki, Pavlos Katonis

**Affiliations:** 1 Department of Orthopaedic and Traumatology, University Hospital of Heraklion, 71110, Crete, Greece; 2 Department of Pathology, Medical School, University of Crete, Heraklion, 71003, Greece

## Abstract

**Background:**

Chronic expanding haematoma (CEH) is a very infrequent event with imprecise developmental mechanism and is rarely reported in literature.

**Case Presentation:**

We present a case of enormous and spontaneous chronic haematoma of the back, expanded from the lower thoracic area to the sacral area, in a young patient without any history of trauma or chronic coagulopathy.

**Conclusion:**

The MRI scan is very useful in preoperative diagnosis, however only the histopathological examination is able to perform the differential diagnosis with soft tissue tumors. Careful surgical treatment is important to minimize the haematoma recurrence.

## Introduction

Chronic expanding haematoma is a rare condition and constitutes a challenge for the physicians because of the difficulty of early and correct diagnosis. The incidence and the true mechanism of this lesion are unknown. They are supposed to be structurally identical with subdural haematomas.

Many case reports have reported CEH in various anatomical regions such as: head, chest, abdominal cavity and extremities with variable sizes. Both MRI scan and histopathological examination are very useful in differential diagnosis with the soft tissue sarcomas. We present herein, a case of impressive spontaneous, chronic expanding haematoma, in a young man without history of trauma or any disease involving the coagulate system function.

## Case Presentation

A 24-year-old man was presented in our department with a large, painless swelling in the lumbar region with a moderate extension to the lower thoracic and to the upper sacral area. In fact, the parents observed the last three months a protuberant mass in this region enlarging gradually. The patient initially was negative to a medical examination. The recent history was negative. The patient was an amateur soccer player, playing with a frequency of 1-2 matches per week. The recent history was unremarkable for trauma or any disease involving the liver and the coagulation tests. The recent pharmacological history for administration of anticoagulant therapy resulted negative.

**Figure 1:**
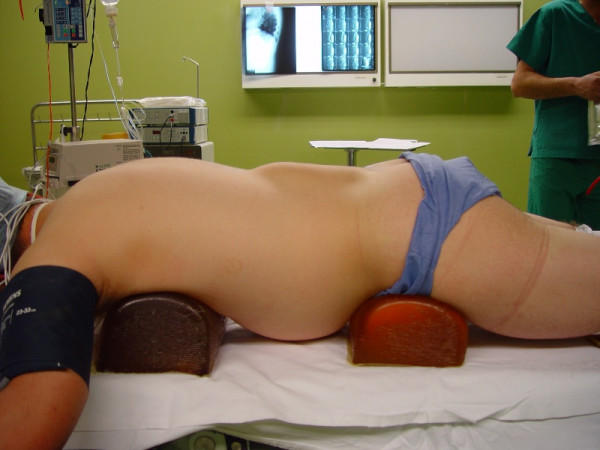
**General aspect of the patient, in prone position on a surgical table**. The swelling area is well demarcated.

**Figure 2:**
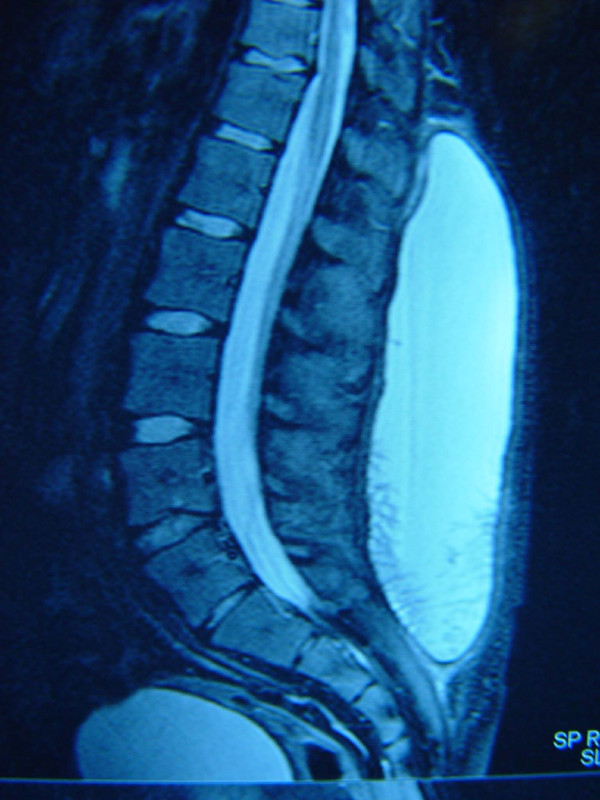
**Sagittal T2-weighted MRI scan demonstrating a large homogenous and expanded haematoma of the lumbar region**.

The inspection revealed a large mass (28 cmx10 cm) from the sacral spine to the lower thoracic region without skin changes. The lesion was soft, fluctuant and painless in palpation (fig 1). The examination of the abdomen, pelvis and the spine was normal. The neurological and the vascular examination of the lower leg resulted normal. The blood tests were done with the red blood, platelets, clotting factors and hepatic function resulting normal. The MRI examination demonstrated an enormous soft tissue mass with features compatible to haematoma. The intraspinal and the extraspinal structures of the lumbar region resulted to be intact (fig. 2).

**Figure 3:**
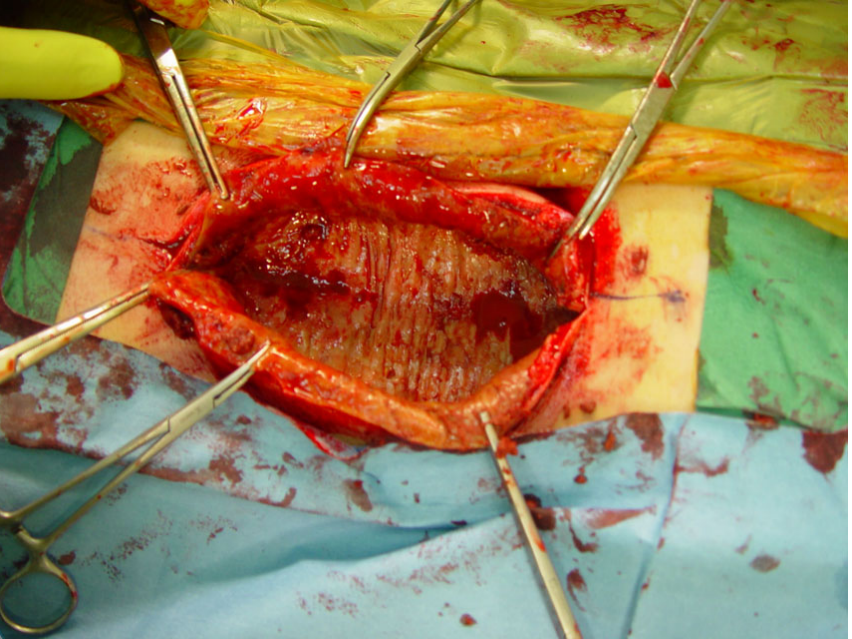
**Intraoperative view of the lesion demostrating the fibrotic wall, after liquid evacuation**.

**Figure 4:**
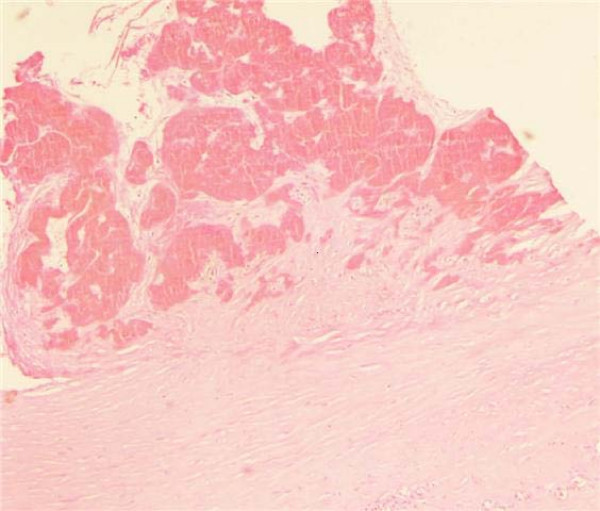
**Hematoxylin and Eosin stain, magnification × 40**. The upper 2/3 of the picture shows a part of hematoma with features of organization and new blood vessels formation. The lower 1/3 of the picture represents hematoma's thick fibrous wall.

We decide to perform a surgical intervention, knowing that, in case of a large chronic haematoma the aspiration is useless because of the high possibility of recurrence. Intraoperatively, we evacuated 2L of altered blood and the findings were compatible with a large soft-tissue fibrous cavity containing a considerable quantity of altered blood clot, fibrous and granulation tissue (fig 3). A complete resection of the fibrotic wall was performed. The underlying fascia was extendedly sutured with the subcutaneous tissue, in order to avoid any dead space where new haematoma can develop. The liquid and soft-tissue culture was sterile. The histopathological examination revealed abundant fibrous tissue with features of recent hemorrhage (fig 4). Two years after the surgical treatment the patient has not shown any sign of recurrence.

## Discussion

According to Reid et al, haematoma that persists and increases in size more than a month after the initial hemorrhagic event are named chronic expanding haematoma [1]. In the majority of cases, soft tissue haematomas resolve spontaneously. The exact etiology of CEHs is not completely known but, they have been developed in regions of previous trauma and surgery, in patients with hemorrhagic diathesis and under anticoagulant therapy, or they can occur spontaneously [2-6]. In our patient we can speculate that, the initial event had been probably so an important but not appreciable sport injury in the lumbar region. To our knowledge spontaneous and so enormous haematoma of the lumbar region has not been reported before in English literature. Late and large haematoma of the lumbar region is a rare event. In a MEDLINE search under "spontaneous expanding haematoma," we found only two articles. Marquardt et al. refer a spontaneous haematoma of the iliac muscle treated conservatively [6]. Cebesoy et al. describe a spontaneous giant expanding haematoma mimicking a soft tissue neoplasm over the lateral left thigh [7].

Mikic has reported several acute immediate posttraumatic haematomas in the lumbo-sacral region, emphasing in their surgical treatment [8]. Case reports and small case series have reported various anatomic locations of CEH from the finger to the head, with greater frequency in the peritronchateric region and the femur [8-10].

Many authors suggest that, the specific etiologic mechanism probably involves a shearing force which detaches the skin and the subcutaneous fascia from the underlying fascia, creating a potential space for the formation of the haematoma [2,3]. The blood, the breakdown products and the vasoactive substances, act synergistically leading in a persistent augmented capillary permeability and haematoma expansion.

The location of CEH in abdominal or thoracic cavity could be accompanied by compression to the adjacent organs and anatomical structures causing serious consequences like hydronefrosis, dispnea, or bone erosion when the haematoma is located respectively in the retroperitoneal space, thorax and femur [2,5,10].

MRI is the most important examination for the preoperative diagnosis. In general, heterogeneous low and high signal intensity is showed respectively on T1 and T2-weighted imaging. Not so rarely, a peripheral rim of low intensity corresponding to the pseudocapsule of hyaline fibrous tissue is detected [2,5]. The presence of new capillaries and the granulation tissue can be shown if contrast material is used. But, the gadolinium may not give additional information differentiating haematoma from the soft tissue sarcomas [2,11]. Some authors suggest the use of the arteriography, in order to exclude an ingoing hemorrhagic event [11].

The differential diagnosis of the CEHs includes hemorrhagic soft tissue tumor (hemangiopericitoma, cavernous hemangioma), sarcoma, actinomycosis and inflammatory pseudotumor [1,2,5]. In very late post-traumatic haematoma, when peripheral calcification has occurred, the differential diagnosis includes myositis ossificans and calcific myonecrosis [12,13].

The final diagnosis is made by the histopathological examination. Microscopically the capsule is consisted in hyalinized fibrous tissue and a granulation tissue with new capillaries and inflammatory cells.

The correct treatment for the CEH is only surgical. Aspiration of the liquid, drainage and curettage could result in serious bleeding and have a higher possibility for recurrence. Complete resection of the capsula and a meticulous suture of the subcutaneous tissue, with the underlying fascia in order to eliminate the dead space are highly recommended [5,6,9].

We conclude, that a chronic expanding haematoma could occur spontaneously or may be caused by a minor or not appreciable trauma. MRI scan is very important in the preoperative diagnosis and the histopathological examination is crucial in the differential diagnosis with the soft tissue sarcomas. A careful preoperative plan and a meticulous surgical treatment could limit considerably the possibility for recurrence.

## Consent

Written informed consent was obtained from the patient for publication of this case report and accompanying images. A copy of the written consent is available for review by the Editor-in-Chief of this journal.

## Competing interests

The authors declare that they have no competing interests.

## Authors' contributions

All authors read and approved the final manuscript.

DP, AB and PK initiated and co-wrote the paper and performed the surgical treatment of the haematoma. KA performed the proof editing. EL examined the specimen and prepared the histological illustrations.
